# Efficacy of a repeat testing protocol for cognitive fatigue assessment: a preliminary study in postconcussive syndrome participants

**DOI:** 10.2217/cnc-2017-0002

**Published:** 2017-12-20

**Authors:** Thomas F Rau, Sarjubhai A Patel, Erik E Guzik, Edmond Sorich, Alan J Pearce

**Affiliations:** 1 The Neural Injury Center, The University of Montana, 32 Campus Drive, Missoula, MT 59812, USA; 2 Department of Biomedical & Pharmaceutical Sciences, The University of Montana Western, 208 Business & Technology Building, 710 S Atlantic St, Dillon, MT 59725, USA; 3 GLIA Diagnostics, PO Box 138N, Armadale, VIC 3143, Australia; 4 Department of Rehabilitation, Nutrition & Sport, La Trobe University, Bundoora, Melbourne, VIC 3086, Australia; 5 Melbourne School of Health Sciences, The University of Melbourne, Parkville, VIC 3010, Australia

**Keywords:** brain concussion, mild cognitive impairment, postconcussion syndrome

## Abstract

**Aim:**

A small but notable number of individuals who suffer a concussion report ongoing cognitive difficulties. This preliminary study investigated the efficacy of repetitive test application to discern cognitive impairment in those with ongoing symptoms.

**Methods:**

Participants (n = 17) with continuing self-reported symptoms following a concussion (∼9 months postinjury) were compared with 17 age group matched controls for working memory and word-list learning.

**Results:**

Both groups performed similarly after the first trial for both assessments. However, in subsequent trials, the postconcussion group performed significantly worse than controls.

**Discussion:**

While further studies to understand the mechanisms are warranted, data from this preliminary study suggest that a repetitive test application may be useful to discern cognitive fatigue in individuals who report ongoing concerns following a concussion.

Mild traumatic brain injury (mTBI) is the most common form of brain injury in the USA [1]. Concussion has been described as a subset of mTBI [2] and is characterized, in the absence of pathological injury, by a rapid onset of nonspecific symptoms and impairment of neurological functions that usually resolve, in the majority of individuals, within 7–10 days [2]. However, there appears to be a small but notable population (∼10%) of concussed individuals that suffer from persistent lingering effects that may last from weeks to many months, which has been recognized as postconcussive syndrome (PCS) [3,4].

PCS generally refers to persistent symptoms beyond the expected recovery period following the injury of 7–10 days. Similar to concussion, there is a constellation of symptoms that have been ascribed to this condition. These include: headaches, vertigo/dizziness, irritability, emotional labiality or irritability, cognitive difficulty (e.g., concentration), sleep disturbance and/or depression and anxiety [5].

Fatigue is also a common symptom, which may be less recognized by clinicians [6]. While fatigue can be discussed in terms of a single construct, in other words, fatigue being either physical or mental; cognitive fatigue, or the inability to perform cognitive tasks for extended periods without a break, has significant impact on the individual with PCS. Activities such as reading a book or communicating with colleagues that may be considered as requiring little demand for healthy people (but actually involves an array of tasks to be performed simultaneously) can be quite difficult for people with PCS. In longitudinal studies, the frequency of prolonged fatigue, usually self-reported complaints of being continually feeling tired [7], in postconcussed individuals varies from 16 to 73% [7–10] of the sample studied. Moreover, there is no correlation between PCS fatigue and severity of the initial injury, age of the person at the time of the injury or time since the injury [6,11].

For the general physician, who may be the first point of contact for people with ongoing symptoms, the issue of prolonged PCS can be difficult to assess as generally the severity of most symptoms declines to pretrauma levels, along with normal neurocognitive testing scores [12,13]. Therefore, the patient may appear ‘normal’. Similarly, patients’ responses to self-reporting scales are open to subjective interpretation. While studies have shown that individuals with severe PCS may perform worse in cognitive tests [6,14,15], those with cognitive fatigue complaints have shown decreased processing and impaired ability to maintain attention during testing [14], leading to frustration by the patient and potentially being misrepresented as the individual having a low tolerance completing such task [16]. No study to date has investigated PCS from a repetitive task performance perspective to elicit the effect of cognitive fatigue, particularly in those who do not present with overt signs of cognitive impairment.

To investigate the efficacy of repetitive application of established tests on cognitive performance, in this preliminary study, we chose two well-known tasks, word-list learning test and working memory span [17], in a group of individuals who had previously suffered a concussion (>6 months) and reported to have continuing symptoms of PCS. We hypothesized that postconcussed individuals would perform worse in subsequent trials compared with healthy, age-matched controls.

## Methods

Using a between groups repeated measures design, a convenience sample of 34 participants (29.9 ± 4.8 years) was recruited for the study from the university population. Recruitment of participants was undertaken after local public announcements, via the print and local television media, highlighting the study and calling for volunteers, follow-up public presentations at the university where there is a large sporting and military population attending classes and local distribution of study flyers across the university and local metropolitan area to sporting clubs and veteran associations. General inclusion criteria for all participants were to be over the age of 18 years, and at least 1 year of postsecondary education (i.e., second year university undergraduate studies). Specific inclusion for PCS required participants to be a minimum of 6 months [18] (mean of 9.3 ± 3.3 months) post ‘impact’ concussion injury (no skull fracture, blast or overpressure brain injury) that was diagnosed by a medical professional. While the International Statistical Classification of Diseases and Related Health Problems and Diagnostic and Statistical Manual of Mental Disorders define PCS at 3 months, we chose 6 months in an attempt to select a concussion group that experienced chronic persistent symptoms. Further, as part of the inclusion criteria, all recruited PCS individuals were required to have no clear diagnosis of impairment related to their concussion following clinical and neuropsychological assessment and had medical clearance to undertake all daily activities. Inclusion criteria for control participants were to report no history of concussion injury. Specific inclusion criteria for control participants required no history of a head injury. Eight PCS participants were excluded, as they did not meet the inclusion criteria (see below). One participant had a congenital neurological condition, two had reported skull fractures and five had experienced a moderate-to-severe traumatic brain injury. Testing was conducted with the understanding and prior written consent of all participants. All recruitment and testing protocols were approved by the University Institutional Review Board and completed according to the principles outlined by the Declaration of Helsinki.

**Figure 1.**
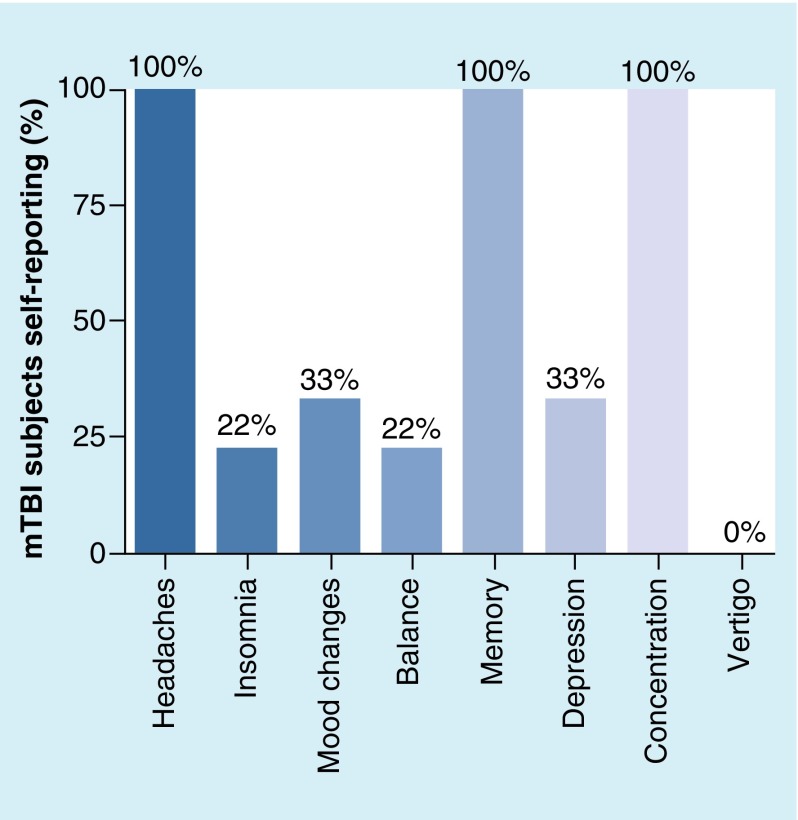
**Self-reported symptoms from the mild traumatic brain injury group.**

Included participants (PCS: n = 17; 7 female; 28.9 ± 4.5 years and control participants: n = 17; 9 female; 30.9 ± 5.1) were instructed to abstain from drug and/or alcohol use for a minimum of 24 h prior to testing. Participants completed a basic questionnaire regarding their concussion history, which included time since last concussion, etiology of their concussion (falls: n = 6; motor vehicle/bicycle/skateboard impact: n = 8; violence: n = 3) and if they had more than one concussion (80% of the participants reported more than one event). When reporting concussion history, PCS participants recalled a loss of consciousness <30 min, <24 h of post-traumatic amnesia and a Glasgow Coma Scale of 13–15. Participants also listed self-reported ongoing concerns (Figure 1).

### Working memory

Working memory is an essential component of executive function that encompasses three basic processes [19]: first, the access of stored information; second, ‘online’ operations on this information; and third, a motor output response based upon the first and second processes. The working memory span task in this study utilized the commercially available Simon^®^ game (Hasbro, RI, USA), which has been used previously by Gendle and Ransom [20] to quickly and efficiently assess working memory span in adults. The game itself is a 12.5 cm × 9.5 cm oval with green, red, blue and yellow plastic buttons paired with distinct tones. For each random sequence presented, the buttons in the sequence illuminate in turn paired with a specific tone to match the color. Sequences start at one and end at a possible maximum sequence of 50, with successfully recalled sequence increased by one until an error is made, terminating the test. Gendle and Ransom have demonstrated that the game is resistant to habituation, practice effects and/or proactive interference [20].

Following previously described protocols demonstrating reliability and validity in working memory span using the Simon game as a proxy working memory assessment [20], all participants completed three trials with 60 s rest between trials. To ensure thorough understanding of the testing, participants were read a standardized script (Supplementary File 1) [20]. To further ensure comprehension of the assessment, the test administrator demonstrated the procedure for the participant, performing a five-sequence span as a demonstration prior to participant testing. The last span achieved (out of maximum of 50) was recorded by the test administrator.

### Word-list learning test

The word-list test provides an assessment of cognitive strategies and processes involved in both learning and remembering verbal information [21]. While there are multiple variations of word-list learning tests, generally the test can assess multiple aspects of verbal memory including the amount of material learned [22]. Previous studies have shown that word-list learning tests were sensitive to the effects of mild cognitive impairment [23–25].

The word-list learning test was a modification of the traditional testing protocol [21], that included the immediate recall, but without the 20 min delayed recall or recognition components [21]. Specifically, this protocol involved participants reading out aloud and memorizing the list of words (Supplementary File 2) with a time limit of 15 s before handing the list back to the test administrator. Participants then began recalling as many words as possible. Once the individual recalled as many words as they remembered (out of a maximum of 17), they verbally confirmed by saying ‘done’. Three trials were completed (with the participant allowed to read the words for 15 s at the start of each trial) with a 60 s rest between each trial. For all of the words, singular and plural tenses were required to be recalled correctly. For example, recalling ‘car’ when the word was ‘cars’ did not count as a correct recall.

### Statistical analysis

All data were prescreened using Shapiro–Wilk (SW) tests and found to be normally distributed (SW: 0.88–0.94; p > 0.05). Statistical analyses were performed using SPSS V24 (SPSS, Inc., USA). Data were analyzed using repeated measures ANOVA followed by post hoc comparisons with Bonferonni adjustment. Pearson correlation co-efficient was employed to explore a relation between performance and the number and time since concussion in the PCS group. Descriptive data are presented as group means (± standard deviation). Cohen's *d* effect size was calculated by the difference between two means divided by the mean of the standard deviation [26]. Alpha was set at p < 0.05.

## Results

### Working memory

**Figure 2.**
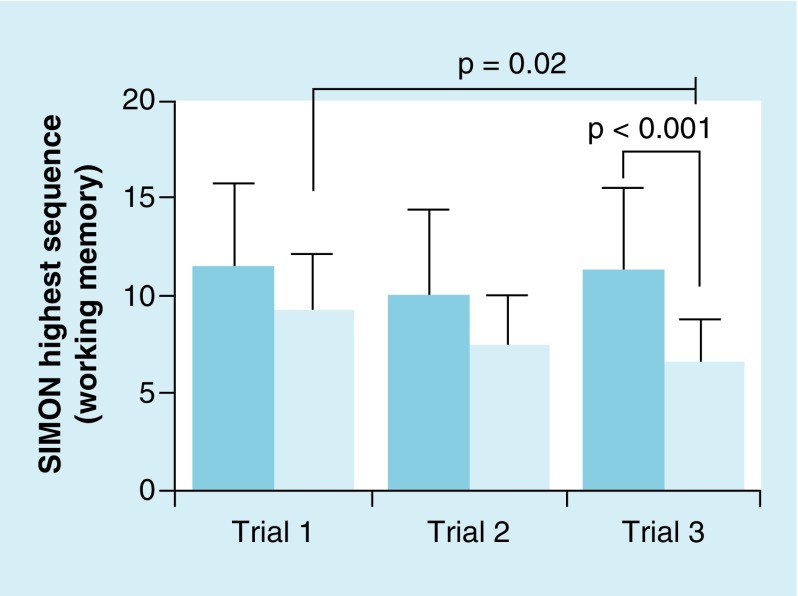
**Mean (± standard deviation) SIMON^®^ game performance (highest successful sequence) between mild traumatic brain injury group (darker blue) and healthy controls (light blue).** While the control group maintained performance, the mild traumatic brain injury group showed a significant worsening of performance from trial 1 to 3 (p = 0.02). No differences were observed between groups for trials 1 and 2; trial 3 showed a significant difference between the groups (p < 0.001).

Figure 2 illustrates the working memory performance. ANOVA revealed no interaction for group by time (*F*_2,31_ = 2.02; p = 0.14), but significant main effects for time (*F*_2,31_ = 3.63; p = 0.04) and group (*F*_1,32_ = 16.08; p < 0.001). Post hoc analyses showed the concussion group performed worse from time points 1 to 3 (*F*_2,27_ = 4.46; p = 0.02). No significant differences were observed between groups in time 1 (*F*_1,32_ = 2.97; p = 0.09; *d* = 0.59) and time 2 (*F*_1,32_ = 1.94; p = 0.17; *d* = 0.48). However, time 3 showed significant difference between the groups (*F*_1,32_ = 15.35; p < 0.001; *d* = 1.34).

No interaction (*F*_2,30_ = 1.42; p = 0.26) or main effects (time: *F*_2,30_ = 1.49; p = 0.24 and group: *F*_1,15_ = 0.05; p = 0.82) were observed in performance between participants with single and multiple concussions. No significant correlations were found in the PCS group between working memory performance decrements and the number of concussions reported (*r* = -0.06; p = 0.83), or the time since last concussion (*r* = -0.19; p = 0.45).

### Word-list learning

**Figure 3.**
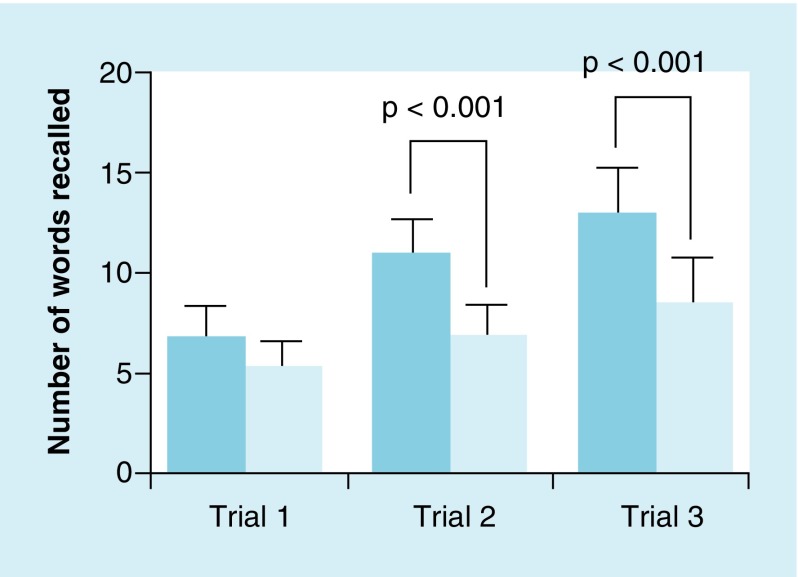
**Mean (± standard deviation) word recall performance between mild traumatic brain injury group (darker blue) and healthy controls (light bule).** Both groups significantly increased over each subsequent trial (p < 0.001). Comparing the groups, no significant difference was found between the groups after trial 1 (p = 0.13). However, significant differences in recalling words were observed between groups after trials 2 (p < 0.001) and 3 (p < 0.001).

Word-list learning test performance is shown in Figure 3. A significant interaction effect for recall was found for group by time (*F*_2,41_ = 27.21; p < 0.001). Both groups improved significantly over each time point (control: *F*_2,26_ = 137.71; p < 0.001 and concussion: *F*_2,30_ = 18.28; p < 0.001). Post hoc comparisons between groups at each time point showed no differences after time 1 (*F*_1,32_ = 2.47; p = 0.13; *d* = 0.53); however, significant differences were found between groups after time 2 (*F*_1,32_ = 50.85; p < 0.001; *d* = 1.88) and time 3 (*F*_1,32_ = 30.56; p < 0.001; *d* = 1.91).

No interaction (*F*_2,30_ = 0.91; p = 0.88) or main effect for group (*F*_1,15_ = 0.06; p = 0.81) was observed in performance between participants with single and multiple concussions. There was a significant effect for time (*F*_1.7,26_ = 13.49; p < 0.001). *Post hoc* analyses with Bonferroni adjustment showed significant improvements observed in the third time point compared with the time 1 (p = 0.002) and time 2 (p = 0.003), for both the single and multiple concussed participants. However, no significant correlations were found in the PCS group between word-list learning performance decrements and the number of concussions reported (*r* = 0.23; p = 0.36), or the time since last concussion (*r* = -0.06; p = 0.81).

## Discussion

To the best of our knowledge, this preliminary study is the first report that using established tests for working memory span and word-list learning, in a repetitive fashion, could discern cognitive performance in our group of individuals reporting with PCS following a concussion >6 months [18]. Compared with healthy controls that maintained consistent results in working memory, the PCS group showed no differences after the first trial, but significant decrements with repeated performance. Similarly, no differences were found between groups after the first trial of the word-list learning. Subsequent trials found improvement in both groups; however, the rate of improvement was significantly greater in the control group compared with the PCS group. The findings of this preliminary study suggest that using a repetitive test design can elucidate decreased cognitive performance in individuals who may have otherwise performed comparably to controls traditional testing. Using repetitive testing protocol, taking between 8 and 12 min, can assist the general physician, who may be the first person a PCS patient approaches to determine if indeed the patient requires further assessment from a neurologist or neuropsychologist.

In our testing, we observed that those who had a history of concussions performed worse with repetitive testing irrespective of concussion history (i.e., self-reporting single and multiple concussions). It was also interesting to note that the concussion group participants (both single and multiple concussions), while being actively engaged during the entire testing session, expressing visible frustration in completing the tasks repeatedly that was not present in the control individuals. Although it has previously been suggested that frustration may be due to low tolerance for ‘boring tasks’ [16], it may also be argued that this visible frustration stems from a diminished information processing capacity associated with incomplete recovery within specific brain areas. While the exact cause of moderated cognitive performance is not known [6], there have been several models to predict the mechanisms for poorer performance when undertaking prolonged cognitive tasks with PCS. It may be that this reduced processing capacity is due to either reduced metabolic capacity [15] or abnormal functioning of underlying neural networks [27]. For example, Peskind *et al*. [28] demonstrated regional hypometabolism in infratentorial (cerebellum, vermis and pons) and medial temporal brain regions in military veterans with multiple episodes of blast-related mTBI. More recently, Arenth *et al*. [29] observed in post-mTBI individuals (1.7 years postinjury) decreased white matter integrity in corpus callosum correlating to reduced cognitive performance. Conversely, while it has been suggested that fatigue can impact cognitive functioning [9], leading to frustration, little research has investigated the pathophysiological mechanisms underlying memory and, more generally, mental fatigue following recovery from mTBI/concussion [7]. Alternatively, other studies have suggested engagement in more extensive neural networks in people with PCS, compared with healthy controls, when completing similar mental tasks [15,30,31], suggesting abnormally increased cerebral effort after brain injury [6], leading to reduced performance more rapidly.

Given the novel nature of this preliminary study, several limitations require acknowledgement. Gendle and Ransom [20] admit that the SIMON game is a less ‘pure’ measure of working memory span than classic assessments such as the Knox Cube Test or the Corsi Block Tapping Task, where the blocks are all of the same color. However, the authors assert that the SIMON game, characterized by colored buttons and unique auditory tones associated with each sequence, serves as a mnemonic aids during memory recall of the pattern [20]. Therefore, healthy participants could obtain high span scores through sustained attention of both color and/or tone patterns, creating an opportunity to differentiate controls from individuals who have suffered a previous concussion and continue to report ongoing symptoms.

With the PCS participant group, the issue of self-reporting is a limitation, not only in this study but also in concussion studies involving individuals with ongoing issues following concussion event(s) [32,33]. Further, 80% reported having a history of multiple concussions, suggesting that the effects we measured were maybe linked to repeated injury. Although, it should be noted that the 20% that had been exposed to a single concussion did not perform differently from the individuals that had suffered repeated concussions. Further we found no correlation between performance decrements for either the working memory or the word-list learning tasks. However, based on the relatively small size of our cohort, meaningful conclusions about the effect of a single versus repetitive concussion on the cognitive performance tests should be interpreted with caution, with further studies comparing single versus repeated concussion histories.

We also acknowledge where individual's self-reporting ongoing post-traumatic complaints volunteer for the study, the issue of self-selection bias cannot be unnoticed. Despite this, our data present no differences in trial 1, followed by statistically significant differences, and large effect sizes in trials 2 and 3 of both assessments suggest that the data from this study demonstrate that using a repeated testing paradigm, as opposed to the traditional single application of an assessment can distinguish cognitive performance differences between PCS and controls. Certainly, future studies should aim to recruit postconcussion individuals who do not have any ongoing issues (asymptomatic), and compare to symptomatic PCS individuals with ongoing symptoms. Further, we also suggest collecting data regarding fatigue/cognitive fatigue in everyday situations from participant's significant others (e.g., family, partner) will assist findings. Finally, it may be argued that the differences between groups may be due to floor effects. As no differences were found after the first trial, but only after trials 2 and 3, we consider these novel findings as important to investigate further, along with other domains of cognition, in future studies.

Future studies should also incorporate specific cognitive fatigue screening. Pre- and postvisual analog scales for pre- and post-testing fatigue can be administered on the day, while the mental fatigue scale [6], a 15-question survey that incorporates affective, cognitive and physical symptoms, sleep duration and daytime fatigue severity variations, has been shown to significantly correlate (*r* = -0.37) to information processing. Further studies should look to incorporate the mental fatigue scale with our repetitive testing protocols, particularly if there is disparity between the individual's self-report complaints and the clinical assessment (being medically cleared for daily activities).

## Conclusion

This preliminary study demonstrates the feasibility of a rapid assessment protocol measuring cognitive performance for participants who may have ongoing post-traumatic complaints, but have not been given a clear diagnosis of impairment related to their concussion previously. Using multiple, back-to-back trials of established cognitive tests in a single testing session, can complement current assessment protocols, particularly with individuals with self-reported prolonged concerns following their concussion.

**Executive summary**

- A small postconcussive syndrome (PCS) generally refers to persistent symptoms beyond the expected recovery period; and similar to concussion, there is a constellation of symptoms that have been ascribed to this condition.
- Whilstindividuals with PCS may appear ‘normal’ and perform satisfactorily withtraditional cognitive testing, the inability to maintain concentration and attention, leading to frustration.
- The aim of this preliminary study was to elucidate decrements in cognitiveperformance in individuals with PCS.

**Methods**

- Seventeen individuals with PCS (mean 9.3 months) were compared to 17 age matched controlswith no history of concussion with two tests; working memory and word-listlearning.
- Unliketraditional testing, we applied each task consecutively three times withminimal rest between tasks.

**Results**

- For both cognitive tasks there were no differences between groups after trial 1. However significant differences were observed in trials 2 and 3.
- The working memory task showed a significant decrement in performance in the PCS group compared to the controls.
- Controls showed significant improvement in the word-list learning task, whilst the PCS showed no improvement.

**Discussion**

- This preliminary study has shown that using traditional testing protocols in arepetitive manner can elucidate cognitive performance decrements that are notusually seen after one trial.
- Further studies are required to investigate if similar patterns are found using a repetitive protocol for tests of cognition.
- However for clinicians, who may be the first professional an individual with ongoing PCS will consult, applying these two simple tests in a repetitive capacity will allow for rapid screening for PCS that can take less than 10 minutes.

## References

[1] Faul MXL, Wald MM, Coronado VG (2010). Centers for Disease Control. Traumatic brain injury in the United States: emergency department visits, hospitalizations and deaths 2002–2006. http://www.cdc.gov/traumaticbraininjury/pdf/blue_book.pdf.

[2] Dimou S, Lagopoulos J (2014). Toward objective markers of concussion in sport: a review of white matter and neurometabolic changes in the brain after sports-related concussion. *J. Neurotrauma*.

[3] Organization WH (1992). *The ICD-10 Classification of Mental and Behavioural Disorders: Clinical Descriptions and Diagnostic Guidelines*.

[4] Ryan LM, Warden DL (2003). Post concussion syndrome. *Int. Rev. Psychiatry*.

[5] Broshek DK, De Marco AP, Freeman JR (2015). A review of post-concussion syndrome and psychological factors associated with concussion. *Brain Inj.*.

[6] Johansson B, Rönnbäck L (2014). Long-lasting mental fatigue after traumatic brain injury – a major problem most often neglected diagnostic criteria, assessment, relation to emotional and cognitive problems, cellular background, and aspects on treatment. *Traumatic Brain Injury*.

[7] Johansson B, Rönnbäck L, Agrawal A (2012). Mental fatigue; a common long term consequence after a brain injury. *Brain Injury – Functional Aspects, Rehabilitation and Prevention*.

[8] Stulemeijer M, van der Werf S, Bleijenberg G, Biert J, Brauer J, Vos PE (2006). Recovery from mild traumatic brain injury: a focus on fatigue. *J. Neurol.*.

[9] van der Naalt J, van Zomeren A, Sluiter W, Minderhoud J (1999). One year outcome in mild to moderate head injury: the predictive value of acute injury characteristics related to complaints and return to work. *J. Neurol. Neurosurg. Psychiatry*.

[10] O'Connor C, Colantonio A, Polatajko H (2005). Long term symptoms and limitations of activity of people with traumatic brain injury: a ten-year follow-up. *Psychol. Rep.*.

[11] Belmont A, Agar N, Hugeron C, Gallais B, Azouvi P (2006). Fatigue and traumatic brain injury. *Ann. Phys. Rehabil. Med.*.

[12] French LM, Lange RT, Brickell T (2014). Subjective cognitive complaints and neuropsychological test performance following military-related traumatic brain injury. *J. Rehabil. Res. Dev.*.

[13] de Kruijk J, Leffers P, Menheere P, Meerhoff S, Rutten J, Twijnstra A (2002). Prediction of post-traumatic complaints after mild traumatic brain injury: early symptoms and biochemical markers. *J. Neurol. Neurosurg. Psychiatry*.

[14] Johansson B, Berglund P, Rönnbäck L (2009). Mental fatigue and impaired information processing after mild and moderate traumatic brain injury. *Brain Inj.*.

[15] Azouvi P, Couillet J, Leclercq M, Martin Y, Asloun S, Rousseaux M (2004). Divided attention and mental effort after severe traumatic brain injury. *Neuropsychologia*.

[16] Mateer CA, Sira CS (2006). Cognitive and emotional consequences of TBI: intervention strategies for vocational rehabilitation. *NeuroRehabilitation*.

[17] Baddeley A (1996). The fractionation of working memory. *Proc. Natl Acad. Sci. USA*.

[18] Nordin LE, Möller MC, Julin P, Bartfai A, Hashim F, Li T-Q (2016). Post mTBI fatigue is associated with abnormal brain functional connectivity. *Sci. Rep.*.

[19] Goldman-Rakic PS, Plum F (1987). Circuitry of primate prefrontal cortex and regulation of behavior by representational memory. *Handbook of Physiology: Sec. 1. The Nervous System, Vol. 5, Higher Functions of the Brain (Part 1)*.

[20] Gendle MH, Ransom MR (2006). Use of the electronic game Simon as a measure of working memory span in college age adults. *J. Behav. Neurosci. Res.*.

[21] Baek MJ, Kim HJ, Kim S (2012). Comparison between the story recall test and the word-list learning test in Korean patients with mild cognitive impairment and early stage of Alzheimer's disease. *J. Clin. Exp. Neuropsychol.*.

[22] Albert M, Moss M (1992). *Neuropsychology of Memory*.

[23] Karrasch M, Sinervä E, Grönholm P, Rinne J, Laine M (2005). CERAD test performances in amnestic mild cognitive impairment and Alzheimer's disease. *Acta Neurol. Scand.*.

[24] Petersen RC, Smith GE, Waring SC, Ivnik RJ, Tangalos EG, Kokmen E (1999). Mild cognitive impairment: clinical characterization and outcome. *Arch. Neurol.*.

[25] Rabin LA, Paré N, Saykin AJ (2009). Differential memory test sensitivity for diagnosing amnestic mild cognitive impairment and predicting conversion to Alzheimer's disease. *Neuropsychol. Dev. Cogn. B Aging Neuropsychol. Cogn.*.

[26] Cohen J (1988). *Statistical Power Analysis for the Behavioral Sciences*.

[27] Rönnbäck L, Hansson E (2004). On the potential role of glutamate transport in mental fatigue. *J. Neuroinflamm.*.

[28] Peskind ER, Petrie EC, Cross DJ (2011). Cerebrocerebellar hypometabolism associated with repetitive blast exposure mild traumatic brain injury in 12 Iraq war veterans with persistent post-concussive symptoms. *Neuroimage*.

[29] Arenth PM, Russell KC, Scanlon JM, Kessler LJ, Ricker JH (2014). Corpus callosum integrity and neuropsychological performance after traumatic brain injury: a diffusion tensor imaging study. *J. Head Trauma Rehabil.*.

[30] van Zomeren A, van den Burg W (1985). Residual complaints of patients two years after severe head injury. *J. Neurol. Neurosurg. Psychiatry*.

[31] Kohl AD, Wylie G, Genova H, Hillary F, Deluca J (2009). The neural correlates of cognitive fatigue in traumatic brain injury using functional MRI. *Brain Inj.*.

[32] Pearce AJ, Hoy K, Rogers MA (2014). The long-term effects of sports concussion on retired Australian football players: a study using transcranial magnetic stimulation. *J. Neurotrauma*.

[33] Kerr ZY, Marshall SW, Guskiewicz KM (2012). Reliability of concussion history in former professional football players. *Med. Sci. Sports Exercise*.

