# Corrigendum

**DOI:** 10.2217/cnc-2017-0002c1

**Published:** 2018-01-24

In the Preliminary Communication by Thomas F Rau, Sarjubhai A Patel, Erik E Guzik, Edmond Sorich and Alan J Pearce ‘Efficacy of a repeat testing protocol for cognitive fatigue assessment: a preliminary study in postconcussive syndrome participants’, which appeared in the December 2017 issue of *Concussion* 2(4), doi:org/10.2217/cnc-2017-0002 (2017), in Figures 2 & 3 the colors of the bars indicating participant group were inverted. The corrected figures are shown below.

**Figure 2.**
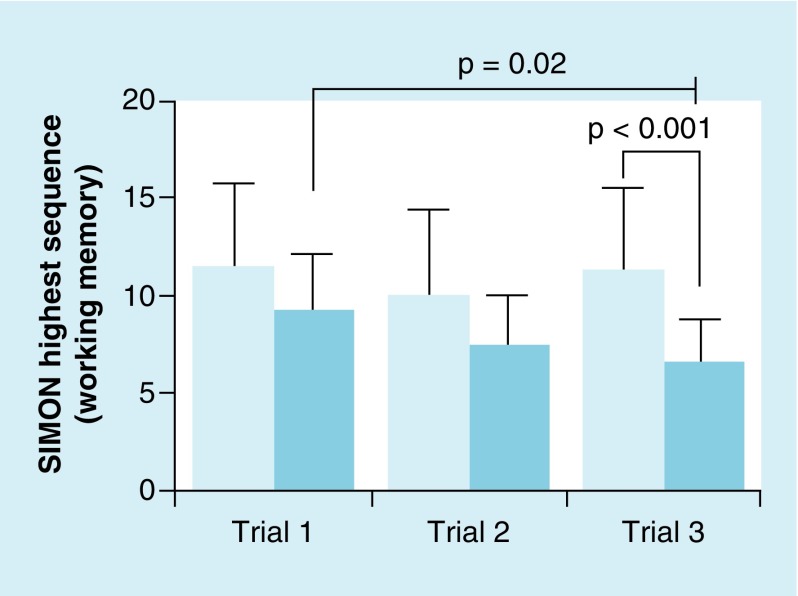
**Mean (± standard deviation) SIMON^®^ game performance (highest successful sequence) between mild traumatic brain injury group (darker blue) and healthy controls (light blue).** While the control group maintained performance, the mild traumatic brain injury group showed a significant worsening of performance from trial 1 to 3 (p = 0.02). No differences were observed between groups for trials 1 and 2; trial 3 showed a significant difference betweenthe groups (p < 0.001).

**Figure 3.**
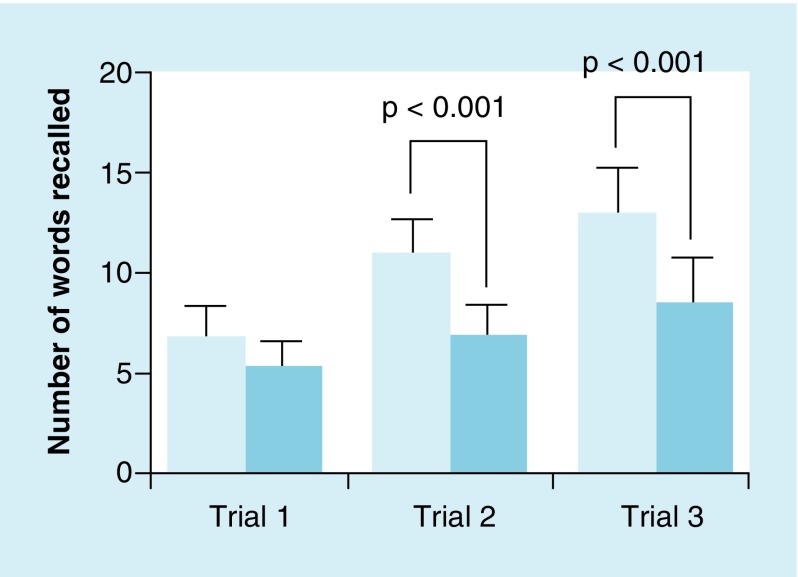
**Mean (± standard deviation) SIMON^®^ game performance (highest successful sequence) between mild traumatic brain injury group (darker blue) and healthy controls (light blue).** While the control group maintained performance, the mild traumatic brain injury group showed a significant worsening of performance from trial 1 to 3 (p = 0.02). No differences were observed between groups for trials1 and 2; trial 3 showed a significant difference betweenthe groups (p < 0.001).

The authors and editors of *Concussion* would like to sincerely apologize for any inconvenience or confusion this may have caused our readers.

