# Supplementary material for: Efficacy of a repeat testing protocol for cognitive fatigue assessment: a preliminary study in postconcussive syndrome participants
Source: Concussion. 2017 Dec 20;2(4):CNC44. doi: 10.2217/cnc-2017-0002 (PMC6122690; doi:10.2217/cnc-2017-0002)
Supplement: Supplementary file 1 [file cnc-02-46-s1.docx]

Supplementary file 1

To ensure thorough understanding of the testing, the following script, developed by Gendle and Ransom [20] was used for each subject: “*This is a simple electronic game that we will use to test memory function. A series of sequences of increasing length will be presented; you must copy each of them in turn. Please work quickly, as you will have only three seconds to respond after a sequence is given. If you make a mistake, a tone will sound and the trial will be over. Do you understand these instructions? Clarification was then provided as needed.”*
