# Supplementary material for: Efficacy of a repeat testing protocol for cognitive fatigue assessment: a preliminary study in postconcussive syndrome participants
Source: Concussion. 2017 Dec 20;2(4):CNC44. doi: 10.2217/cnc-2017-0002 (PMC6122690; doi:10.2217/cnc-2017-0002)
Supplement: Supplementary file 2 [file cnc-02-46-s2.docx]

Supplementary file 2

Cat

House

Fear

Slipped

Birdhouse

Dream

Cars

Bread

Wander

Deliveryman

Desert

Glass

Addition

Laughter

Fire

Rainbow

Splinter
